# Unusual Intron Conservation near Tissue-Regulated Exons Found by Splicing Microarrays

**DOI:** 10.1371/journal.pcbi.0020004

**Published:** 2006-01-20

**Authors:** Charles W Sugnet, Karpagam Srinivasan, Tyson A Clark, Georgeann O'Brien, Melissa S Cline, Hui Wang, Alan Williams, David Kulp, John E Blume, David Haussler, Manuel Ares

**Affiliations:** 1 Department of Computer Science, Baskin School of Engineering, University of California Santa Cruz, Santa Cruz, California, United States of America; 2 Department of Molecular, Cell, and Developmental Biology, Sinsheimer Labs, University of California Santa Cruz, Santa Cruz, California, United States of America; 3 Hughes Undergraduate Research Laboratory, Thimann Laboratories, University of California Santa Cruz, Santa Cruz, California, United States of America; 4 Affymetrix, Santa Clara, California, United States of America; University of California Berkeley, United States of America

## Abstract

Alternative splicing contributes to both gene regulation and protein diversity. To discover broad relationships between regulation of alternative splicing and sequence conservation, we applied a systems approach, using oligonucleotide microarrays designed to capture splicing information across the mouse genome. In a set of 22 adult tissues, we observe differential expression of RNA containing at least two alternative splice junctions for about 40% of the 6,216 alternative events we could detect. Statistical comparisons identify 171 cassette exons whose inclusion or skipping is different in brain relative to other tissues and another 28 exons whose splicing is different in muscle. A subset of these exons is associated with unusual blocks of intron sequence whose conservation in vertebrates rivals that of protein-coding exons. By focusing on sets of exons with similar regulatory patterns, we have identified new sequence motifs implicated in brain and muscle splicing regulation. Of note is a motif that is strikingly similar to the branchpoint consensus but is located downstream of the 5′ splice site of exons included in muscle. Analysis of three paralogous membrane-associated guanylate kinase genes reveals that each contains a paralogous tissue-regulated exon with a similar tissue inclusion pattern. While the intron sequences flanking these exons remain highly conserved among mammalian orthologs, the paralogous flanking intron sequences have diverged considerably, suggesting unusually complex evolution of the regulation of alternative splicing in multigene families.

## Synopsis

Alternative splicing expands the protein-coding potential of genes and genomes. RNAs copied from a gene can be spliced differently to produce distinct proteins under regulatory influences that arise during development or upon environmental change. These authors present a global analysis of alternative splicing in the mouse, using microarray measurements of splicing from 22 adult tissues. The ability to measure thousands of splicing events across the genome in many tissues has allowed the capture of co-regulated sets of exons whose inclusion in mRNA occurs preferentially in a given set of tissues. An examination of the sequences associated with exons whose expression is regulated in brain or muscle as compared to other tissues reveals extreme conservation of intron sequences nearby the regulated exon. These conserved regions contain sequence motifs likely to contribute to the regulation of alternative splicing in brain and muscle cells. The availability of global gene expression data with splicing level resolution should spur the development of computational methods for detecting and predicting alternative splicing and its regulation. In addition, the authors make strong predictions for biological experiments leading to the identification of components and their mechanisms of action in the regulation of splicing during mammalian development.

## Introduction

Splicing is an essential process that constructs protein coding messenger RNA (mRNA) sequences using tiny segments of information buried in the much larger primary transcripts of the eukaryotic gene. Regulated alternative splicing can create different protein coding sequences under different biological circumstances, allowing the production of functionally related but distinct proteins (for review, see [1]). In addition, alternative splicing can mediate the repression of gene expression by stimulating the formation of transcripts subject to nonsense-mediated decay [2–5]. Splicing patterns seem distinct in the vertebrate nervous system compared to other tissues [6,7], and it is tempting to hypothesize that neural alternative splicing contributed to the rapid evolution of the vertebrate brain without large increases in gene number [8].

Biochemical analysis of alternative splicing has shown that numerous RNA binding proteins influence the use of specific splice sites to stimulate splicing events that lead to particular mRNA isoforms [1,9]. These RNA binding proteins may activate or repress the use of splice sites by binding to nearby sequences in the exon (exonic splicing enhancers [ESEs] or exonic splicing silencers [ESSs]) or in the intron (intronic splicing enhancers [ISEs] or intronic splicing silencers [ISSs] [1,9]). In many cases, multiple RNA binding proteins combine to create repressing and activating influences that produce patterns of splicing control [6,9]. Some proteins, such as SR proteins and the CELF proteins, have mostly activating roles, whereas others, such as hnRNP A1, PTB, and nPTB, have mostly repressing roles. Certain proteins can either activate or repress splicing in different contexts, depending on the position of their binding sites or the expression of other RNA binding proteins [10,11].

A complete catalog of the RNA sequences corresponding to the enhancers and silencers bound by splicing regulatory proteins would greatly aid the understanding of splicing regulatory networks. Thus far, there are only a handful of splicing regulators whose corresponding RNA binding motifs have been identified (for review, see [12]), whereas there may be many splicing regulators among the hundreds of RNA binding proteins encoded by the mouse genome. In addition, several related but distinct genes produce proteins that bind the same or overlapping sets of sequences; for example, Fox-1 and RBM9 each bind UGCAUG [13,14], and the branchpoint binding protein SF1 and the protein quaking (QK) each bind UACUAAC-like motifs [15–17]. Adding to this complexity is the tendency for the mRNAs of RNA binding proteins to be alternatively spliced, leading to multiple RNA binding protein isoforms with potentially different functions (e.g., [14,18–20]). Currently, the methods available for expanding the list of known regulators and their target sequences are limited, and the development of this catalog is in the early stage [12].

Much of the available genomic information on alternative splicing is derived by the alignment of large numbers of expressed sequence tags (ESTs) and messenger RNAs (high-quality cDNA sequences) to genome sequences (for many genomes, see [21]). The analysis of exons that appear to be constitutive (i.e., present in every example of a transcript from a given locus) or alternative (exons or parts of exons that are sometimes skipped) has led to the successful identification of many distinguishing features of alternatively spliced regions [22–28], even allowing their accurate prediction without cDNA evidence [26,29,30]. Although cDNA libraries have been invaluable for discovering general features of alternatively spliced exons, it is difficult to connect specific regulatory sequences to specific biological conditions with confidence due to variable and sometimes missing information about the source materials and methods of cDNA library construction. The relatively low number of transcripts present from any one gene also makes it difficult to estimate differences in expression levels using library representation as a measure. Thus, more direct methods are needed to associate alternative splicing events with underlying biological conditions.

The recent application of microarray technology to questions of splicing and splicing regulation promises to reveal parallel connections between many splicing events and specific biological or experimental conditions [31–41]. Analysis of experimental changes in splicing for many genes simultaneously should reveal biological conditions necessary for proper splicing regulation in a way that analysis of cDNA libraries cannot, and with breadth that cannot be achieved by analysis of a reporter construct or a few endogenous target genes. To demonstrate this, we constructed a DNA microarray designed to capture splicing information for about 6,200 alternative events in the mouse transcriptome, using a combination of splice junction and exon probes, and have hybridized RNA from 22 adult mouse tissues. We examine splicing in these tissues by asking three questions.

First we ask, Which RNA isoforms are present in a particular tissue sample? To answer this simple question, we used a new method based on comparing the intensity of the probes in a probe set to the distribution of intensities from all probes with similar G + C level. This is similar in spirit although different in approach to the present-absent calls from Affymetrix MAS 5.0 algorithms [42], as this microarray did not contain mismatch probes. Using RT-PCR, we show that this method has a true-positive rate of 85%.

Second we ask, Which RNA isoforms are differentially expressed across the tissues examined? For each RNA isoform, the intensities of the isoform-specific junction probes were examined across tissues using the Kruskal-Wallis statistical test. After correcting for multiple testing, about 40% of the 6,216 total alternative splicing events examined were found to have more than one RNA form that was differentially expressed, indicating widespread tissue differences in splicing over the tissues.

Third we ask, Which cassette exons are included differentially between brain (or muscle) and nonbrain (or nonmuscle) tissues? To answer this, we used a regression-based bootstrapping method, which also allows an estimate of the relative change in skipping and inclusion in the two sample groups. We analyzed the intron sequences associated with exon skipping events that are differentially regulated in brain or muscle relative to other tissues and found unusual patterns of sequence conservation that provide new information about tissue regulation of alternative splicing and its evolution.

## Results

### Broad Detection of Tissue-Regulated Alternative Splicing in Mouse Using Microarrays

**Figure 1:**
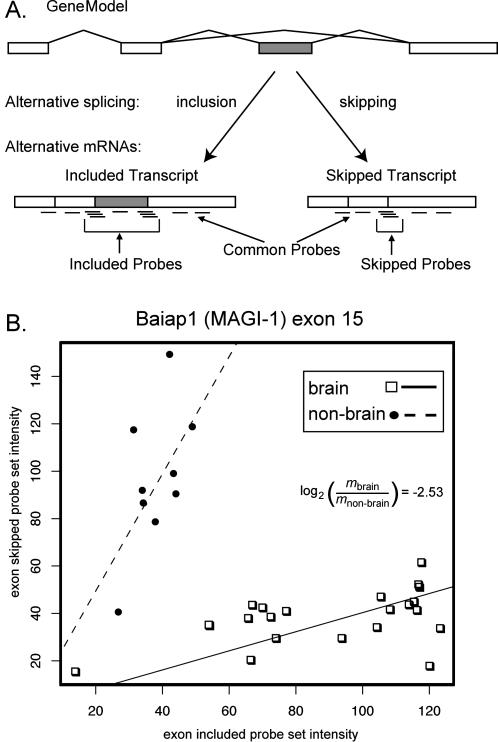
Array Design and Analysis of Splicing-Sensitive Microarray Data (A) Probe design and expression counts of alternative event-specific probe sets. Probe sets were designed to both the skipping splice junctions and include splice junctions, as well as the alternative exons when possible, ensuring that probe sets are specific to the exon skipped or included spliced isoforms. Probe sets for constitutive portions of the gene were used to measure overall expression of the locus. (B) Scatterplot of skip probe set intensity versus include probe set intensity for Biaip exon 15 in brain (squares) and nonbrain (circles) tissue samples. Each data point is derived from one RNA sample and represents the skip-to-include ratio for that sample. The lines represent the robust regression coefficient (constrained to go through the origin) for each tissue group. The log_2_ difference in the slopes is −2.53, indicating 5.7-fold inclusion in brain relative to nonbrain tissues.

The general idea of using DNA microarrays with combinations of splice junction and exon probes designed to capture splicing information has been presented previously [31,35] and applied in various forms by a number of groups to questions of alternative splicing [32–34,37,38,40]. The specific DNA microarray designed for these experiments uses the Affymetrix format [43,44] similar to the microarray used previously [35], except that most mismatch probes were not included. Briefly, for each gene there is a “common” probe set to determine whether the gene is expressed, as well as “isoform-specific” splice junction and exon probe sets able to distinguish alternative mRNA isoforms (Figure 1A). Each splice junction probe set contains six 25-mer DNA oligonucleotide probes tiled across the junction (see Materials and Methods).

As the junction probes should be specific for the RNA form derived by the alternative splicing event that creates the junction, we first asked simply whether each particular junction is detected in different tissues. To do this, we first determined which genes were likely to be expressed above background, using the “common” probe set designed to detect gene expression (Figure 1A). For the alternative junction probe sets in the genes that were expressed, we then estimated the probability that the intensity measured by each junction probe was above background, using an empirical cumulative distribution function (CDF) (see Materials and Methods). Thus, to detect the expression of a particular alternatively spliced RNA from a particular gene, we demand that both the gene probe set and the junction-specific probe set be called expressed. Alternative splicing is then inferred if two alternative isoforms are both significantly expressed.

In the set of adult mouse tissues we studied, a large number and variety of alternative splicing events were detected using this first method (Table S1). Among the class of expressed genes for which we could detect at least one alternative event, we could observe a second alternative event in 18% to 30% of cases, depending on the type of splicing event. Alternatively skipped (cassette) exons were the largest class, with 376 exons. For the purposes of this study, we defined a cassette exon to be any exon that can be included or skipped in its entirety, regardless of other alternative splicing events that affect it (e.g., alternative 3′ or 5′ splice sites). RT-PCR validation experiments (Table S2) indicate that the true-positive rate (fraction of situations in which a splice junction predicted to be expressed by the microarray is detected by RT-PCR) is about 85% (217 RT-PCRs; a reaction is one primer pair with cDNA from one tissue, designed to detect two isoforms, or 434 independent splice junction tests [see Materials and Methods]), and the false-negative rate (fraction of times that a splice junction that could not be detected on the microarray is detected by RT-PCR) is about 47%, due to the relatively greater ability of RT-PCR to detect low levels of gene expression (data not shown).

Simple present or absent determinations of RNA forms containing particular splice junctions such as those shown in Table S1 are likely to miss changes in alternative splicing that do not involve large changes in overall transcript level. To improve our detection of smaller-scale changes in alternative splicing, we asked which alternative RNA forms are differentially expressed across tissues. To determine this, we used the Kruskal-Wallis test to determine whether the intensity measured for probes specific for each junction in different samples was likely to come from the same or different distributions. Alternative splicing is again inferred if two alternative RNA forms (differing at alternative junctions) are both significantly differentially expressed (Table 1).

**Table 1:**
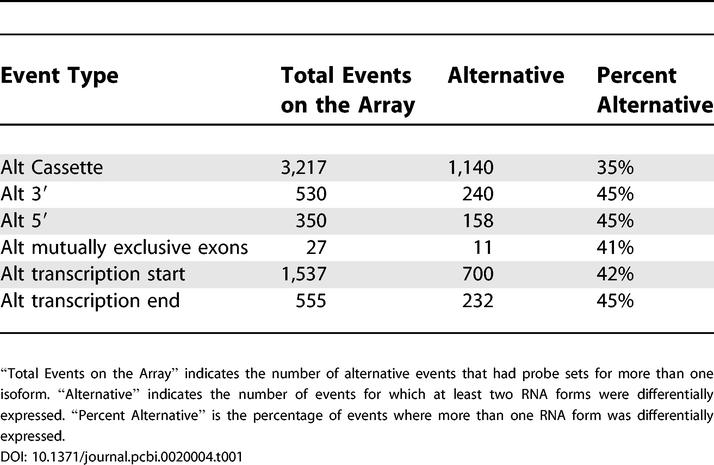
Differential Expression of Alternative Splicing Event Isoforms

For most classes of alternative splicing events, nearly 5-fold more alternative events were identified by the Kruskal-Wallis test than by simple presence-absence tests, as expected. By this analysis, about 40% of the alternative RNA forms we could detect are differentially expressed across tissues. Using the PCR tests for those genes in Table S2 for which at least five tissues were tested, we find that the Kruskal-Wallis test gives an 89% true-positive rate (25 of 28 isoforms predicted to be differentially expressed versus being detected or not detected by nonquantitative RT-PCR across at least five tissues) with a 7% false-positive rate (2 of 28).

These validation data are consistent with other studies using microarrays to detect alternative splicing [32,37]. During validation, evidence for new isoforms not identified in the EST/mRNA data was obtained in about 20% of cases (data not shown), indicating that much alternative splicing remains undiscovered, as other studies have also noted [32]. Since our microarray design relies on initial evidence for alternative splicing from EST/mRNA data, we would not expect our current analysis to detect such events except during validation by RT-PCR. We conclude that, although limited in sensitivity, the data and our analysis are specific and likely provide a conservative representation of splicing events across the adult mouse transcriptome.

### Detection of Strongly Regulated Alternative Splicing in Brain and Muscle Tissues

A critical challenge is to distinguish differences in alternative splicing from changes in transcript level, as the overall transcription of individual genes varies greatly across tissues. To focus on brain- and muscle-enriched alternative splicing independent of changes in transcript level, we devised a simple method to identify pairs of alternative splice junctions whose expression relative to each other differs greatly between two subsets of tissues in our dataset. A measure of alternative splicing is the ratio of skipped isoform to included isoform. We wanted to test whether this ratio is different between two groups of tissues (e.g., brain and nonbrain). A natural measure of the overall ratio is the slope of the line created by plotting the skip probe set intensities versus the include probe set intensities for many samples from a tissue group. If there is a consistent alternative splicing pattern within the group, these values should fall on a line with a slope given by the skip/include ratio (Figure 1B). We then test for differences between the two groups of tissues by bootstrapping multiple rounds of robust regressions for each group and comparing the slopes of the regression lines for the two groups of tissues (Figure 1B; see Materials and Methods). The difference in the slope between the tissue groups is a measure of the difference in the average ratio of skipping to inclusion for the indicated exon in the two different groups of tissues over a wide range of transcription levels. In the example shown, exon 15 of the membrane-associated guanylate kinase (MAGUK) gene Baiap1 (MAGI-1) is preferentially included more than 4-fold in brain tissues compared to the nonbrain tissues in which the gene is expressed (Figure 1B).

By searching this way through all the cassette exons within expressed genes (as defined by intensity measured by “common” probe sets), we identified 171 exons that appear differentially regulated in brain tissue compared to nonbrain tissue (Table S3). Of these, 91 are preferentially skipped in brain, whereas 80 are preferentially included in brain. To focus our studies on exons whose regulation is most extreme between the two groups of tissues, we further examined the set where the log_2_ difference between regression slopes was greater than 2. This criterion resulted in a set of 36 brain-included and 36 brain-skipped cassette exons whose skip/include ratios were more than 4-fold different on the average in brain relative to nonbrain tissues. Details concerning the genes associated with these exons, as well as a set of muscle-regulated exons derived by comparison of heart and skeletal muscle samples with nonmuscle tissues, are found in Tables S3 and S4. Having identified sets of exons with similar levels and patterns of splicing regulation, we compared the nature of their nearby intron sequences to those of a large control set of constitutive exons.

### Evolutionary Conservation of Intron Sequences Adjacent to Tissue-Regulated Exons

**Figure 2:**
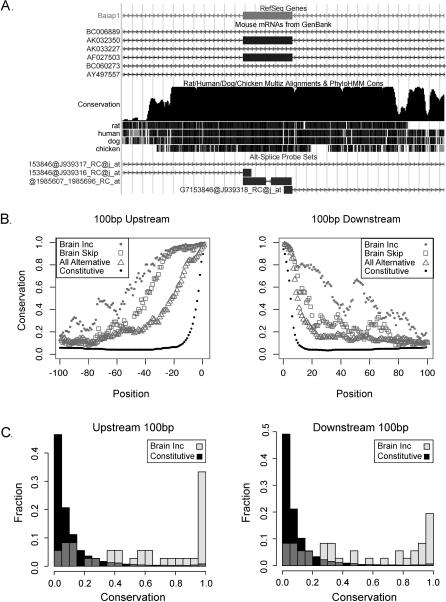
Conservation of Cassette Exons Preferentially Included in Brain (A) Extreme conservation in flanking intronic sequences of Baiap1 cassette exon seen in University of California Santa Cruz genome browser. (B) Median conservation probability at each base 100 base pairs upstream (left) and downstream (right) of the exon for 36 brain-included exons (gray circles), 36 brain-skipped exons (hollow gray squares), about 1,000 skipped mouse exons conserved and alternatively spliced in both human and mouse (gray triangles), and about 47,000 constitutive mouse exons (black circles). These last two sets of exons are from an EST/mRNA-based study [24]. (C) Histograms of the median probability of conservation per 100 base pairs upstream and downstream of the brain preferentially included (light gray), constitutive exons (black), and overlapping regions (dark gray).

A number of studies have noted extended regions of high conservation of intron sequences surrounding ISE and ISS elements found adjacent to regulated exons (e.g., nPTB [45], FGFR1, FGFR2 [46,47]). In addition, EST/mRNA-based studies have noted that alternative exons and their nearby intron regions are generally conserved in different organisms, suggesting the presence of *cis*-acting regulatory elements [22–28]. To examine sequence conservation associated with our brain- or muscle-regulated alternative exons and their nearby introns, we used the program phyloHMM. phyloHMM uses sequence alignments and phylogenetic trees to calculate the posterior probability that an observed alignment results from a conserved rather than a neutral model of evolution [48,49]. Conservation can be compared to the positions of exons using tracks displayed by the University of California Santa Cruz Genome Browser [21]. The brain-included exon 15 of Baiap1 (MAGI-1) is shown as an example (Figure 2A, see also Figure 1B). In addition to conservation of the exon, it is clear that intron sequences adjacent to the exon are highly conserved. Most of the alternative exons identified as brain- or muscle-regulated are associated with conserved adjacent intron sequences (Figure 2B and 2C).

We systematically analyzed this conservation in our set of brain-regulated exons compared to constitutive exons in two ways. First, we asked about the probability of conservation at each nucleotide position (Figure 2B) as distance from the exon increases upstream (left) or downstream (right). Both the brain-included (gray circles) and the brain-skipped (gray squares) exons are significantly more likely to be associated with conserved intron sequences than about 47,000 constitutive mouse exons (black circles, [24]). The conservation levels for about 1,000 unselected skipped exons from mouse that are also skipped in the human transcriptome (gray triangles, [24]) are lower than those of the extremely regulated brain exons. This result suggests that extraction of splicing events by tissue-regulated pattern and magnitude of regulation using arrays also extracts associated sequence conservation patterns. Intron nucleotides near brain-included exons are even more conserved than those that are preferentially skipped (Figure 2B). The probability of conservation generally decreases with distance from the exon although not smoothly. There appear to be “bumps” centered about 50 nucleotides downstream of the 5′ splice site and about 70 nucleotides upstream of the 3′ splice site, suggesting that many brain-included and brain-skipped exons are more likely to have more conserved nucleotides in these regions (Figure 2B). In general, the 3′ splice site region upstream of the regulated exon is more extensively conserved for a longer distance than the region downstream of the exon adjacent to the 5′ splice site (Figure 2B).

Second, we asked about the median probability of conservation of the entire set of 100 nucleotides immediately upstream (Figure 2C, left) or downstream (right) for the brain-included exons and plotted the distribution of their median conservation probabilities (gray bars) compared to the constitutive exons (Figure 2C, black bars). The histograms show that about 30% of the exons in the brain-included set have upstream intron sequences (3′ splice site regions) whose median probability of conservation exceeds 0.9 (left) compared to less than 1% of constitutive exons. About 20% are similarly conserved for the downstream (5′ splice site) region (right). In some cases, the conservation of the intronic regions exceeds that of the exon. Other studies have noted this trend using alternative exons observed in cDNA libraries [24–26]. Our results using microarrays now allow extraction of tissue regulation-associated intron conservation for further analysis (see below).

We were concerned that conservation levels similar to protein coding sequence might indicate that many exons in our set had nearby splice sites that could be used to create larger, protein-coding alternative versions of the exon through splicing. If true, the high level of conservation could be explained by protein coding rather than a splicing-related function, despite the absence of evidence for such splicing in the EST/mRNA data. To test this, we used the program QRNA [50] to analyze the sequences. QRNA examines pairwise alignments of orthologous sequences from different organisms (in this case, mouse and human), notes the pattern of sequence divergence, and evaluates three models of evolutionary constraint: protein coding, RNA structure, and “other.” Using the conserved elements that overlap the brain-included exon as chosen by phyloHMM [48], usually including the exons themselves, QRNA predicts that its RNA structure evolution model fits the data best in 43% of the regions, whereas the protein coding model fits only 19% of these regions. In contrast, for 500 conserved regions that overlap constitutive exons, which usually include only protein-coding sequence, QRNA predicts the RNA structure model for only 16% and chooses the protein-coding model for 69% of the regions. Elimination of the protein-coding model by QRNA for more than 80% of the regions overlapping our regulated set is a strong indication that the conserved sequences associated with regulated exons are unlikely to represent cryptic protein-coding sequence. We conclude that the conservation of intronic sequence is likely due to its function in splicing regulation.

### Searching for Regulatory Motifs in the Conserved Regions near the Tissue-Regulated Alternative Exons

Our sets of exons are defined by similar regulatory patterns obtained from splicing-sensitive microarray data. In contrast to alternative exons culled from unselected EST/mRNA collections in which all regulatory signals are superimposed, the sequence composition of our brain- or muscle-enriched exons may allow identification of ISE and ISS sequences particular to the regulatory events that mediate alternative splicing in these tissues. We wanted to ask whether the conserved regions contain *cis*-acting elements known to be important for splicing regulation. To do this, we examined the frequency of several known splicing regulator RNA-binding motifs in the intronic sequences near the 171 brain-regulated exons to those of a control set of about 47,000 mouse exons that appear constitutively spliced in the mRNA/EST data [24]. To estimate these frequencies, we used the consensus motifs that represent the core elements of more complex recognition sequences. For the regulators PTB and nPTB, we used CUCUCU [51]. For hnRNP H/F, we used GGGGG [52]. The consensus sequence used for the Fox-1 family of proteins A2BP1 and RBM9 was GCAUG [53], and for Nova, it was UCAUY [54]. For hnRNP A1, the consensus motif of UAGGG was used [55]. The approach of using these simple representative sequences for frequency determination estimates is conservative, and it may miss related sequence examples of the motif that nonetheless are functional.

**Figure 3:**
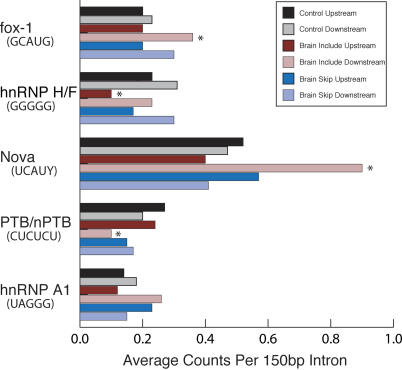
Counts of RNA Binding Motifs in Intron Sequences adjacent to Brain-Regulated Exons The 100 base pairs upstream and downstream regions for constitutive exons (control), preferentially brain-included (brain include), and preferentially brain-skipped exons (brain skip) were evaluated for the presence of sequences related to binding sites for known splicing regulators. Sequences used as the consensus binding sites were Nova: UCAUU or UCAUC; Fox-1: GCAUG; PTB/nPTB: CUCUCU; hnRNP-H/F: GGGGG; and hnRNP A1: UAGGG.

When we compared the frequency of these consensus sequences near our selected sets of exons to the frequency to those of the constitutive exons (Figure 3), we found that both the Nova and the Fox-1 consensus motifs were enriched significantly in the 150 nucleotides of intron sequence downstream of brain-included exons (*P* = 3.9 × 10^−7^ and 5.3 × 10^−6^, respectively), whereas no such enrichment was observed downstream of the brain-skipped exons (Figure 3). We observed an enrichment of Fox-1 sites upstream of exons skipped in muscle, as did Jin et al. [53]; however, too few observations were made to estimate the statistical significance of this finding. These data support the idea that these two splicing factors contribute to the inclusion of nearby exons in the brain from positions downstream of the regulated exon. Nova is expressed only in neural tissues and can activate or repress exon inclusion [10,56–58], in some cases positively from downstream positions [56,58]. Sequences containing the Fox-1 motif have also been shown to activate inclusion of exons from this position [53,59–62], although proteins that bind it are not restricted to brain [13,14,53,60,61].

In contrast, the PTB/nPTB consensus sequence is significantly depleted (*P* = 4.7 × 10^−3^) from the region downstream of the brain-included exons (Figure 3). Consensus hnRNP F/H binding sites are significantly depleted (*P* = 2.6 × 10^−3^) from the region upstream of the brain-included exons (Figure 3). This suggests that the absence of PTB/nPTB binding to the region downstream of the exon, or absence of hnRNP F/H binding to the region upstream of the exon, may be important for proper regulation of some brain-included exons. hnRNP A1 binding sites did not appear significantly enriched or depleted in either region near the brain-included exons (*P* > 0.05). Although we have restricted our search to the 150 nucleotides proximal to the upstream and downstream sides of the regulated exons, we find significant enrichment and depletion of intronic sequence motifs known to influence alternative splicing. We conclude that selection of alternative splicing events on the basis of microarray data results in the identification of new candidates for exons regulated in the brain by RNA binding proteins with Nova and Fox-1 type specificities.

**Figure 4:**
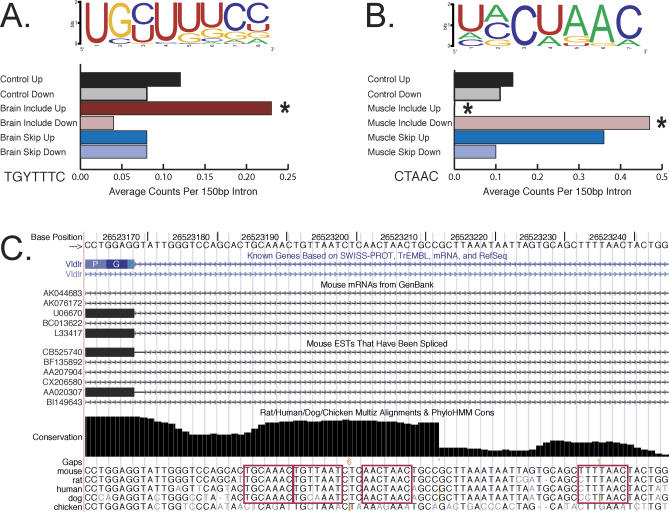
New Motifs in the Tissue-Regulated Exons (A) A new motif UGYUUUC found upstream of brain-included exons. The logo is shown above, and the graph of the frequency of this motif in different regions of the brain-enriched exons is below. (B) A sequence similar to the recognition sites for SF1 and QK proteins is enriched near the 5′ splice site of the muscle-included exons. The logo is shown above, and the graph of the frequency of the core of this motif in different regions of the muscle-enriched exons is below. (C) Locations of multiple copies of conserved sequences matching the motif in (B) found in a conserved “bump” downstream of a muscle-included (and brain-skipped) exon 16 in the *Vldlr* gene.

In addition to examining the frequency of the consensus sequence of known RNA binding factors, we examined the tissue-regulated exons for novel motifs. To identify new motifs near tissue-regulated exons, we used the Improbizer motif-finding program written by Jim Kent [63]. This program identifies sequence motifs present in a set of sequences compared to a background sequence set. As a background sequence set, we used the upstream or downstream regions of a set of about 47,000 exons showing no alternative splicing in mouse [24]. Improbizer identified the Nova motif in the intron sequence downstream from the brain-included exons, consistent with the increased counts of the Nova core sequence (Figure 3). Two additional interesting motifs were found by Improbizer (Figure 4), as well as by MEME [64,65], although the precise weight matrices of the motifs differ slightly (data not shown). A motif with consensus sequence UGYUUUC (Y = C or U) was identified in the 150 nucleotides upstream of brain-included exons (Figure 4A). Although pyrimidine rich, this motif is found above a background of constitutive exons and thus may not be a typical feature of polypyrimidine tracts generally associated with the 3′ splice site. To estimate the probability of finding this motif by chance, the input sequences were randomly permuted 1,000 times, and the Improbizer program was run for each randomized set. The UGYUUUC motif found in the natural sequences had a higher score than any motif found in the 1,000 randomized control runs. We further examined the counts of all 4-mers in the 150 nucleotides upstream using a binomial test to look for differences in the proportion of 4-mers compared to the control set. Five 4-mers had significant *P*-values after using the Bonferroni correction to account for multiple testing with the significance level of 0.05/256 = 1.95 × 10^−4^. The 4-mers found to be enriched were CUCC, CUCU, CUUU, UCCU, and UGCU. Two of the five significant 4-mers are subsequences of the UGYUUUC motif found by Improbizer, further indicating that this motif is not likely to have occurred by chance. None of the above 4-mers was enriched in the 150 nucleotides upstream of the exons preferentially skipped in brain. This motif could either repress exon inclusion in non-brain tissues or activate inclusion in brain tissues. No splicing factors are known to bind this sequence at this time.

A motif with striking similarity to the branchpoint consensus sequence UACUAAC is found in the intron region downstream of the 5′ splice site of heart and skeletal muscle–included exons (Figure 4B). While the small number of sequences makes testing the statistical significance of finding this motif using Improbizer difficult, the biological importance of this sequence motif has been thoroughly demonstrated in several contexts (see Discussion). To investigate this further, we performed an analysis similar to that of the other known motifs (Figure 3) and measured the frequency of the core of the branchpoint motif (Figure 4B, CUAAC) near muscle exons compared to constitutive exons. We found that CUAAC is enriched downstream of the 5′ splice site in muscle exons but not upstream of the exons compared to constitutive exons. Near some muscle-included exons such as Vldlr exon 16, there are multiple copies of this motif contained within regions that are highly conserved among mammals (Figure 4C). We propose that a protein recognizes this motif and activates inclusion of nearby exons in heart and skeletal muscle. After identifying this motif in the muscle-included exons, we revisited the brain exons and determined the frequency of the core CUAAC sequence in the nearby regions. The frequency of CUAAC is enriched downstream of the brain-included exons as well, whereas exons skipped in brain showed no significant enrichment. This suggests that CUAAC motifs downstream of the 5′ splice site may activate exon inclusion in both muscle and brain cells.

### Paralogous Brain-Included Exons in Three Members of the MAGUK Family

**Figure 5:**
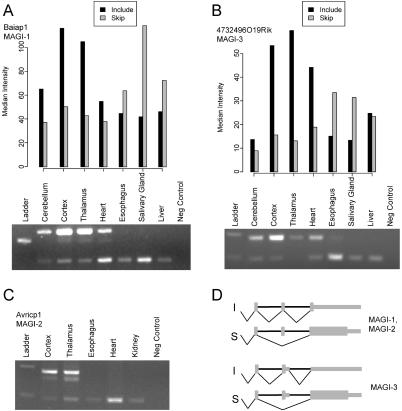
Tissue-Regulated Splicing Controls the C-Terminal Sequences of Mouse MAGI Proteins (A) Microarray intensity (top) and RT-PCR results (bottom) for the alternative exon in Baiap1. (B) Microarray intensity (top) and RT-PCR results (bottom) for the alternative exon in 653047C02Rik. (C) RT-PCR results (bottom) for the alternative exon in Avricp1 (this gene was not present on the array). (D) Diagram of the alternative splicing and coding patterns at the 3′ end of the mouse MAGI genes.

Four of the 22 members of the MAGUK family present in the mouse genome have exons that appear to be differentially included in brain tissues. The guanylate kinases are important in the transport, anchoring, and signaling of synaptic receptors and ion channels (for review, see [66]). The kinase domain no longer functions, and it appears that the MAGUK family has evolved to act as a scaffold to bind other proteins. The four MAGUK family members found to have brain-included exons are called Cask, Dlgh1, Baiap1 (human ortholog is Magi-1), and 4732496O19Rik (human ortholog is Magi-3). The latter two are paralogs apparently resulting from gene duplication, and their regulated alternative exons are also paralogous (Figure 5). Another paralog called Acvrinp1 (human ortholog is Magi-2) also contains a paralogous exon that is alternatively spliced in humans. Although there is no mouse cDNA representing the skipping event, RT-PCR analysis confirmed that the Acvrinp1 exon is also alternatively spliced in mouse, being included in the brain but skipped in nonbrain tissues (Figure 5C). In all three genes, the alternative exon lies just downstream of a C-terminal PDZ domain and is not predicted to influence nonsense-mediated decay, suggesting that the protein encoded by these alternative exons may influence PDZ-mediated protein-protein interactions in the brain (for review, see [67]).

**Figure 6:**
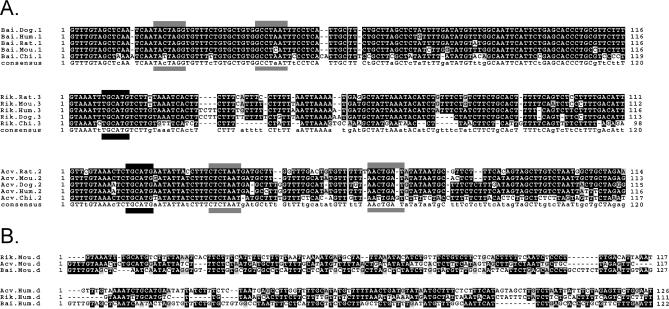
Multiple Alignment of the Flanking Intron Sequences Downstream of the Alternative Exons from Baiap1 (MAGI-1), Acvrinp1 (MAGI-2), and 4732496O19Rik (MAGI-3) and the Orthologous Sequences from Rat, Human, Dog, and Chicken While the orthologous sequences have high conservation between them (A), the paralogous sequences have diverged considerably (B). The 5′ splice sites are at the left. Genome sequences similar to TACTAAC are between gray bars. In the region downstream of the 5′ splice site these may act as regulatory binding sites for SF1, quaking (QK), or some other factor. Fox-1 sites are shown between black bars.

All three of the MAGI gene alternative exons have nearby intronic sequences that are highly conserved with their respective orthologous regions in other organisms (Figure 6A). The 89-nucleotide exon 15 of mouse Baiap1 lies in a highly conserved region (Figure 6A, see also Figure 2A), and there is EST evidence supporting its alternative splicing in species as distant as chicken. The tissue patterns of alternative splicing for these paralogous exons are similar, as detected by the microarray data and confirmed by RT-PCR (Figure 5 and data not shown). The intron sequences downstream from the 5′ splice site are highly conserved in the orthologs (Figure 6A), but comparison of the paralogous intron sequences shows that they have diverged considerably in overall sequence (Figure 6B). The same conservation pattern holds for the exons and the region upstream of the exons. This appears to conflict with the expectation that alternative exons of common descent and regulatory pattern should possess common regulatory elements. This expectation holds for orthologs despite occupying different genomes but apparently not for paralogs sharing similar regulatory profiles within the same genome.

## Discussion

### Identification of Brain and Muscle-Regulated Exons Using Microarrays

A significant challenge of using DNA microarrays to study alternative splicing is separating changes in overall gene-derived transcript level from changes in alternative splicing. An additional difficulty is that splicing cannot be assessed in tissues in which the gene is not expressed, a situation that creates missing data, which in turn can confound many statistical similarity metrics. Numerous groups are now using microarrays to study the regulation of splicing, and all have had to develop methods to account for transcriptional effects [31–41]. Examining the fold change of isoform-specific probes, normalized by the change in probes common to all transcripts, has worked well in treatment and control experimental designs [31,39,41]. More sophisticated model-based methods that either directly estimate isoform concentration [35,38] or find loci that do not fit a constitutive exon model, thus identifying candidate alternative exons [32,68], have been applied to tissue panels to detect the occurrence of alternative splicing differences between the tissues. Other methods have used pairwise anticorrelation to identify cases where a constitutive exon model is inappropriate [37]. By comparing splicing in grouped sets of tissue samples (e.g., brain versus nonbrain), we were able to use a relatively simple regression-based statistical test to identify regulated alternative splicing (Figure 1B). This test isolates our parameter of interest, the relative use of pairs of alternative junctions, and identifies a significant difference in this parameter between two sample populations. Using this method, we discovered hundreds of tissue-regulated alternative splicing events (Tables S3 and S4).

The identification of a splicing event as tissue-regulated does not necessarily imply that all cells within a tissue share the same splicing pattern. Many tissues are heterogeneous populations of distinct cell types, and dramatic examples of differences in splicing in individual cells within a tissue are known (for review, see [69]). Our method appears sufficient to capture splicing events despite this heterogeneity. However, it is possible that many instances of cell type–specific splicing are missed because the superposition of the splicing patterns from the tissue generates a mixed signal. Such cell type–specific splicing events could be identified using purified cell populations from the same tissue.

### Extreme Conservation near Alternative Exons

Large blocks of conserved sequence are found in the introns both upstream and downstream of the alternative exons identified in our microarray data (Figure 2), consistent with earlier computational studies based on EST/mRNA data [24–26]. The high levels of conservation do not appear to be due to cryptic protein-coding function as determined by QRNA, even though the conserved regions analyzed often included a portion of the coding exon. It is possible that these highly conserved regions contain multiple RNA binding protein motifs that act in concert to regulate the splicing of the alternative exon. A selective pressure on the type, number, and order of these RNA binding proteins could explain the large blocks of conserved sequence seen near these exons (for examples and additional references, see [45–47]). It is hard to visualize how such large blocks of conservation are required given the flexible way that RNA protein binding sites can function in other contexts. It seems likely that the secondary RNA structures of these regions play some role in the binding of proteins that influence alternative splicing or may play some direct role themselves by an as-yet-unknown mechanism. It is difficult to make convincing secondary structure models of these conserved regions since they lack the phylogenetic variation necessary to support such models. Much future work remains to be done to determine the functional elements that regulate these alternative exons.

### Conserved Intron Sequences near Regulated Exons Are Enriched for Known Motifs

Nova has previously been shown to regulate alternative splicing in the brain [10,56–58,70], and the enrichment of Nova sites in the brain-included exons extends the potential list of Nova splicing targets. Our data show that the 150 nucleotides downstream of the brain-included exons has a significant increase in Nova-1 consensus motif sites, whereas we could not detect enrichment in the regions immediately upstream of brain-included exons. These results suggest that most often Nova stimulates exon inclusion in the brain from positions in the150 nucleotides downstream of the exon (Figure 3).

Similarly, the consensus motif for Fox-1 proteins is enriched downstream but not upstream of the brain-included exons, suggesting that like Nova-1, Fox-1 proteins contribute most often to activation of exons from positions to the 5′ splice site side (Figure 3). At least one other mouse protein, RBM9, has an RRM family RNA recognition motif essentially identical to that of vertebrate Fox-1 [13,14,53] and is therefore likely to recognize the UGCAUG sequence. Unlike Nova proteins, the expression of Fox-1 proteins is not normally restricted to brain [13,14,53]; thus, the Fox-1 motif is not strictly brain associated and is known to regulate nonneural splicing events [13,14,23,53,59–62,71,72]. Previous EST/mRNA-based studies [23,25,59] have noted an enrichment of the Fox-1 motif near brain-included alternative exons but not the enrichment of the Nova motif. This illustrates the utility of empirical data that accurately enriched our exon set for strong regulation.

### Discovery of New Motifs Associated with Regulated Exons

An unexpected finding is that a sequence motif similar to the yeast branchpoint consensus sequence UACUAAC is found enriched downstream of exons included in heart and skeletal muscle (Figure 4). Although the set of muscle-included exons was too small for robust statistical evaluation of the Improbizer results, this motif is known to be biologically important (see below). In addition, the frequency of occurrence of the core of the motif CUAAC is significantly enriched in this region (Figure 4B). Several proteins have been shown to bind sequences related to this motif. The best understood in molecular terms is SF1, a KH-domain family member that binds the pre-mRNA branchpoint during intron recognition [17,73,74] and is not known to bind and regulate exon inclusion from downstream sites. An NMR structure of the SF1 KH-domain bound to UACUAAC RNA provides a detailed picture of the contacts that lead to sequence specificity [17]. Recent work has revisited the question of whether SF1 is required for splicing of every mammalian intron [75,76], opening the possibility that it might have functions beyond those as a general splicing factor. SF1 is broadly expressed, so if it has a special role in regulating muscle exon inclusion it will be interesting to know how it might carry out both a general and a regulatory function in the same cells. Strikingly, muscle-specific exons appear to be depleted of CUAAC sequences upstream of the intron, suggesting they may have weak SF1-branchpoint interactions.

Other metazoan proteins with similar KH domains that bind nearly identical sequences include Caenorhabditis elegans GLD-1 and the vertebrate QK known to regulate cytoplasmic mRNA stability through sites in the 3′ UTR of their target mRNAs [15,16,77]. QK is expressed in brain, heart, and muscle [20], and one QK isoform (QK-I5) enters the nucleus, where it may regulate splicing [19,78]. Experiments to test the role of QK in splicing regulation clearly show it can influence splicing [18]; however, these studies predated the identification of the QK binding site [15]. Thus, it is unclear whether QK splicing regulation [18] is directly mediated through the UACUAAC-like QK binding site. In addition, the mouse genome contains at least two additional genes *(KHDRBS2* and *KHDRBS3)* predicted to contain KH domains with binding specificities that could be similar to SF1 and QK, although little is known of their expression or localization. Further work will be necessary to determine if and how this sequence influences exon inclusion in heart and skeletal muscle. Although many studies of heart- and muscle-specific splicing have identified motifs and factors using directed analysis of one or a few substrates, none has identified this motif.

We have also identified a novel UGYUUUC motif that is enriched upstream of the exons most strongly included in brain (Figure 4A). Thus far, no RNA binding protein is reported to recognize UGYUUUC. It is interesting to note that whereas exons preferentially included in brain are near intron sequences enriched for known ISE motifs, those preferentially skipped in the brain are not. It is likely that many exons that are preferentially skipped in brain may be preferentially included in other tissues, and their tissue-specific activation signatures are superimposed and thus lost by the heterogeneous grouping of “not brain.” It is important to note that our analysis does not guarantee that every important motif has been identified. We have selected the new motifs we discuss as contrasting examples: one novel with no known biological explanation, and the other with well-demonstrated biological functions in other contexts.

### Conservation in Orthologs and Divergence in Paralogs: Why Maintain Similar Splicing Patterns Using Distinct Sequences?

Nature has provided an interesting case study with the paralogous alternative exons of the MAGUK proteins (Figures 5 and 6). The paralogous alternative exons in the MAGUK genes suggest either that there are many combinations of distinct RNA binding sites that can mediate a similar tissue-specific splicing pattern or that other levels of regulation besides primary RNA sequence exist. The striking difference in the primary sequence of the exons, and nearby intronic sequence, coupled with the apparent similarity in splicing profiles conflicts with the parsimonious hypothesis that alternative exons resulting from a gene duplication event should have similar RNA binding motif profiles regulating them. It is possible that the regulation of the exon occurs through some more distal site as other studies have shown for both Nova [56] and Fox-1 [53,60] or could even reside within the exons themselves. Yet the high level of conservation in orthologous species suggests that the primary sequence of these exons, and nearby introns, is important for the function of these genes. It is also possible that there are subtle differences in the regulation of the paralogous exons that we cannot distinguish looking at the heterogeneous cell populations (e.g., neurons and glia) that make up a tissue extract. Even if there are differences within regulation between subpopulations of cells in brain, it seems plausible to expect that at least the mechanism of skipping in other tissues might be common among them. Taken together, the large blocks of extremely conserved intronic sequence, much larger than typical RNA binding motifs (Figure 2), the QRNA prediction that these blocks are not protein coding, and the paralogous alternative exons with common regulation from divergent sequence (Figures 5 and 6) all suggest that there are other levels of evolutionary constraint on these regions in addition to RNA binding motifs that influence alternative splicing. In addition to the possible direct effect of the primary sequence motifs we have noted, the folded structure of these RNA regions could be important, even though we cannot now determine the nature of these folds. Alternatively, relatively small contributions of the primary sequence to splicing efficiency could make very large contributions to evolutionary fitness and reproduction of vertebrates that could be difficult to assay in the laboratory. It will be important to understand the functional basis for this unusual conservation. A recent study has suggested that evolutionary forces act to produce either multiply duplicated gene families poor in alternative splicing or single genes with complex alternative splicing [8]; however, the MAGUK gene family appears to have taken an intermediate path.

## Materials and Methods

### Design of microarray to monitor alternative splicing in the mouse.

Oligonucleotide microarrays to study mouse alternative splicing were designed as part of this collaboration and constructed by Affymetrix (Santa Clara, California, United States). All cDNA and EST information used for developing the mouse splicing sensitive chips were aligned to the February 2002 genome freeze of the mouse genome. Briefly, mouse cDNA sequences were aligned to the genome using psLayout, an early version of BLAT [79]. Orientation of cDNA was determined using EST read directions, coding sequence annotations, and poly(A) signals. Alignment discontinuities between cDNA and genome bordered in the genome by GT–AG, AT–AC, or GC–AG were interpreted as introns. The program AltMerge [80] was run on the aligned cDNAs to create a gene model that describes the different paths through the gene created by alternative splicing, for each gene.

DNA oligonucleotide probes are synthesized on a glass surface by photolithographic methods [43,44]. Probes used in this work were 25 nucleotides long and restricted to 711 × 711 adjacent positions. Each microarray has 5.05 × 10^6^ probes. Probes are grouped conceptually into “probe sets” of six to ten probes designed to measure the same transcript feature. Three types of probe sets were created for each gene model. One set, the “gene probe set,” consists of eight to ten probes placed in the regions of the gene found in all mRNA isoforms and is meant to measure the overall transcript level from the gene. There are gene probe sets for 15,000 RefSeq genes in addition to those for alternative splicing events in genes not identified in RefSeq. A second kind of probe set is the “splice junction probe set,” which consists of six probes that step across a splice junction, centered at positions −3, −2, −1, +1, +2, and +3 relative to the junction. The third kind of probe set is the “exon probe set,” which consists of variable number of probes for distinct alternative exon regions in the gene model. After design and manufacture using the February 2002 assembly of the mouse genome, the probes were reanalyzed and remapped to the May 2004 assembly. The microarray has probes sufficient for the detection of more than 6,000 alternative splicing events. All probes are “perfect match” probes and there are no “mismatch” probes. Thus, we used alternative data analysis strategies to subtract background nonspecific hybridization signals from the true hybridization signals (described below). A small set of known alternatively spliced genes was annotated by hand, and probe sets designed for these genes included mismatch and perfect match probes.

### Animal care and maintenance.

Male and female 8-wk-old C57/BLJ6 were obtained when necessary from Simonsen Labs (Gilroy, California, United States). Animals were provided with unlimited access to food and water and were housed on site for at least 2 wk before use.

### Tissue dissection.

Tissues were dissected from individual age- and weight-matched mice. Animals were deeply anesthetized with an overdose of Nembutal by intraperitoneal injection prior to any dissection. Brain tissues used were cerebellum, cortex, olfactory bulb, pineal gland, hindbrain, median eminence, thalamus, and cerebellar hemispheres. Nonbrain tissues used were spinal cord, heart, lung, liver, spleen, kidney, testis, ovary, mammary gland, salivary gland, esophagus, stomach, small intestine, colon, and skeletal muscle. Males were used for all tissues, except that estrus cycle–matched females were used for mammary gland and ovary. Tissues from different individuals were not pooled, with the exception of olfactory bulb, pineal gland, testis, and ovary. Tissues were frozen in liquid nitrogen, and RNA was extracted immediately or stored after freezing at −80 °C until RNA extraction.

### RNA extraction.

RNA from cortex, cerebellum, olfactory bulb, thalamus, hindbrain, pineal gland, median eminence, cerebellar hemispheres, lung, liver, spleen, testis, colon, kidney, small intestine, ovaries, mammary gland, and salivary gland was extracted using the Invitrogen Micro-to-Midi Total RNA Purification System (catalog 12183–018; Carlsbad, California, United States). RNA from skeletal muscle, heart, and esophagus was extracted using the Qiagen RNEasy kit for Fibrous Tissue (catalog 74704; Valencia, California, United States). All RNA samples were treated with DNase I for 30 min at 37 °C, extracted twice with phenol/chloroform and once with chloroform, and ethanol precipitated. RNA samples were run on an Agilent Bioanalyzer (Agilent Technologies, Palo Alto, California, United States) to determine RNA integrity as measured by the ratio of 28S to 18S rRNA. RNA concentration was measured using either a regular spectrophotometer or a Nanodrop short–path length spectrophotometer.

### Labeling protocol.

Tissue RNA from at least three individual animals (or three separate groups of animals in the case where tissues needed to be pooled to obtain sufficient RNA) was used to make target for hybridization to triplicate oligonucleotide microarrays. RNA was primed with random hexamers and reverse transcribed. After the reaction was completed, RNA was removed from the reaction by alkaline hydrolysis and the cDNA was purified using Qiagen PCR Quick Purification Kit. A typical reaction started with 5 to 6 μg of total RNA usually yielded about 3 μg of cDNA. The cDNA was then fragmented using DNase I in an empirically controlled reaction that yields DNA fragments of 50 to 200 bases. This fragmented cDNA was then end labeled using terminal deoxynucleotidyl transferase and DNA-Labeling-Reagent-1a (DLR-1a), which is a biotinylated dideoxynucleoside triphosphate. Labeled targets were mixed with Affymetrix Eukaryotic mix (biotin-labeled oligos for which control probes exist on the chip as internal controls), and heated at 99 °C for 5 min before hybridizing to microarray. Targets were hybridized to chips in 7% DMSO solution for 16 h overnight at 50 °C. Microarrays were washed and processed with anti-biotin antibodies and streptavidin-phycoerythrin according to the standard Affymetrix protocol. After scanning the chips and aligning the grids to the scanned image, intensity values were extracted using the software associated with the Affymetrix scanner.

### Normalization and analysis of intensity data.

Normalization and analysis of intensity values from the DNA microarrays were done using a quantile normalization [81], and probe set summaries were derived using the Robust Multi-chip Analysis (RMA) procedure [82,83] with two modifications. The first modification was to remove all probes with 17 or more continuous bases that match to any other mouse transcript, in order to minimize cross-hybridization issues. The second modification was to use the mode of the probe intensity values of similar GC content probes for the background estimate of a particular probe. For example, if a probe has a GC count of 16, then the mode of the intensity of all the probes with a GC count of 16 was used as a background estimate.

### Global detection and comparison of alternative splicing.

The DNA microarrays used in this study lack mismatch probes, preventing use of the standard Affymetrix MAS 5.0 protocols for calling the absence or presence of a target complementary to a particular probe set in the sample [42]. We first removed from consideration all probes with 17 or more continuous bases that spuriously match to any other mouse transcript, in order to minimize cross-hybridization issues. Next the remaining probe intensities for an array were used to construct an empirical CDF. The empirically derived CDF was used to calculate an empirical *P*-value that a particular probe's intensity arose from the background of all probes. This corresponds to using the relative rank of a probe's intensity as a *P*-value for the probability that the probe's intensity is due to background. Probes were stratified by G + C count as probes with a higher G + C count are known to generally have more nonspecific binding due to the thermodynamically more favorable G + C base pairing. For each probe set, the median *P*-value of the set of individual probes in the probe set was used as the *P*-value for that probe set. The probe set *P*-values of expression for biological replicates were combined using Fisher's χ^2^ method [84] to derive a probability of expression in a particular sample. To detect the expression of a particular alternatively spliced RNA from a particular gene, we first determine that the gene is expressed. For each expressed gene, we then determine whether RNA containing either of two or more alternative splice junctions is detectable using the isoform-specific probes according to the method described above. If probe sets for two or more alternative isoforms are called “present” in any sample of the data, this is taken as evidence for alternative splicing. As the probe selection process is constrained at splice junctions and we are using only perfect match probes, rather than both perfect match and mismatch probes, these estimates may not be as robust as those derived from the MAS 5.0 algorithm.

### Estimating overall differential expression of alternatively spliced RNAs across tissues.

For each alternative splicing event, the junction probe sets that would hybridize to individual isoforms were identified and the probes they contained were used for the Kruskal-Wallis test [85]. Individual probe intensity values were normalized as described above, and probe intensities from the replicates from each tissue were grouped together. The Kruskal-Wallis test as implemented by the R function kruskal.test was used to test the hypothesis that the probe intensities come from identical populations. If the resulting *P*-value was small enough, the null hypothesis was rejected and the alternative hypothesis that the probes were differentially expressed across tissues was accepted. To determine an appropriate value for the 0.01 significance level, we first ran 12,740 Kruskal-Wallis tests on randomly selected probe sets and found the α value associated with the 1% quantile of randomly selected probes to be 1.975486 × 10^−3^. To account for multiple testing, a Bonferroni-corrected α value of 1.975486 × 10^−3^/1.2740 × 10^4^ = 1.550617 × 10^−7^ was used as a *P*-value cutoff for significance. If more than one alternative RNA form was differentially expressed, this was taken as evidence of alternative splicing across the tissues. These data are presented in Table 1.

### Capturing strong differential tissue regulation of exon inclusion and skipping.

Regression coefficients between the include and the skip probe set intensities for brain and nonbrain (or heart and skeletal muscle versus nonmuscle) tissues were compared to determine if the level of inclusion or skipping of each exon is different between the two populations of tissues. To avoid assuming normality, a bootstrap method was used to determine the significance of the difference between the regression coefficient for two tissue populations using the method described by Wilcox [85]. Briefly, the number of times that the regression coefficient for one population is greater than the other is tabulated for all of the bootstrap samples and is used to calculate the significance of the difference between the two populations. Formally, we wish to test the null hypothesis that the skip/include ratios are the same in the brain and nonbrain groups, H_0_ : β_brain_ = β_nonbrain_. We resample the data with replacement *D* times. For each resampling *d, I_d_* = 1 if β_brain_ − β_nonbrain_ > 0 and *I_d_* = 0 if β_brain_ − β_nonbrain_ = 0. Thus, P(H_0_) = 2 * min[(Σ_d_ *I/D*), 1 − (Σ_d_ *I/D*)]. We reject the null hypothesis H_0_ if P(H_0_) < 10^−4^. To implement this test, we took 10,000 bootstrap samples from the two populations (*D* = 10,000), and a robust regression coefficient was calculated for each one using iterated re-weighted least squares as implemented by the rlm function in R [86]. Thus, if one group of tissues T_1_ uses the include isoform more than another group of tissues T_2_, then a subsample of T_1_ should also use the include isoform more than a subsample of T_2_. Looking at 10,000 sets of such subsamples strengthens the argument that the differences in skip/include ratios in the two groups are not due to chance. All regression calculations were required to go through the origin (zero intensity), since without transcription there can be no included or skipped transcripts. To help ensure that the genes analyzed had independent evidence for expression before analyzing their splice junction signals, only samples in which the gene probe set was called present (see previous section) with a *P-*value of ≤0.75 were used in the regression calculation. Furthermore, a minimum of eight hybridizations in which the gene was considered expressed for each tissue group was required before a splicing event could be considered for analysis. The difference between regression slopes can be used to estimate the magnitude of the fold difference in splicing between the two groups of tissues. Only events for which all bootstrap regression tests consistently called one slope greater than another (*P* < 0.0001) were used in subsequent analysis.

### Motif finding and calculation of motif enrichment.

To identify new motifs we used Improbizer [63]. As described in the supplement to [63], Improbizer searches for motifs in DNA or RNA sequences that occur with improbable frequency using an algorithm adapted from MEME. The algorithm works with position-weight matrices (PWMs). At each position in a motif, the PWM contains a probability for each of the four nucleotides. The algorithm (1) constructs an initial PWM based on the first 6-mer in the input sequence set, (2) finds the best placement of the PWM on each sequence in the input, (3) constructs a new PWM by taking a weighted average of the PWMs found in step 2 and assesses whether adding or subtracting a column from either end of the PWM will increase the score, (4) repeats steps 2 through 4 until there is no change in the PWM, (5) then constructs a new initial PWM based on the next 6-mer in the input, and (6) repeats steps 2 through 6 over a user-specified subset of the input (by default all of the first 20 input sequences), (7) reports the best scoring PWM, (8) probabilistically erases the occurrences of the best scoring PWM from each sequence, and (9) iterates steps 1 through 8 until it has found as many PWMs as the user has requested. A key to the algorithm is the scoring of PWM placements. The score is an odds score: *P*_observed/PWM_/*P*_observed/background model_ where the numerator is the product of the probabilities of the nucleotides at a particular position according to the PWM, and the denominator is the probability according to a simple Markov chain constructed by examining frequencies of nucleotide occurrences throughout a large background (nonenriched for the motifs of interest) sequence set. Further details and the source code are available from W. J. Kent (kent@soe.ucsc.edu). There is also a Web site (http://www.soe.ucsc.edu/~kent/improbizer/improbizer.html) with an online version of the algorithm.

As a background sequence set, we used about 47,000 exons that show no alternative splicing in mouse [24]. For motif frequency determination, the counts of consensus motifs were calculated for the 150 nucleotides upstream and downstream from the exon of interest. Counts were compared to those of the about 47,000 constitutive exons using Pearson's χ^2^ test statistic, as implemented by the R function prop.test, to determine the likelihood (*P-*value) that the motif counts of interest could have come from the same distribution found in the constitutive exons.

### RT-PCR validation of microarray predictions.

cDNA was generated from about 2 μg of total RNA using the TaqMan Reverse Transcription Reagents kit (Applied Biosciences, Foster City, California, United States) using a mixture of oligo-dT and random hexamers, following the manufacturer's instructions. For PCR, about 50 to 100 ng of cDNA was used as a template with primer pairs designed to amplify the region containing the skipped exon. Reactions used Taq Polymerase (Promega, Madison, Wisconsin, United States) and were run for 25 to 35 cycles at annealing temperatures appropriate for the primer pairs used. PCR products were run out on 2% agarose gels and stained with ethidium bromide.

## Supporting Information

Table S1 Presence Absence Determination of Alternative RNA Forms (22 KB PDF)

Table S2 RT-PCR Testing of Array Predictions (22 KB PDF)

Table S3 Brain-Regulated Exons (23 KB PDF)

Table S4 Muscle-Regulated Exons (15 KB PDF)

### Accession Numbers

Information about the microarray data in this study has been deposited at GEO (http://www.ncbi.nlm.nih.gov/geo) under the accession number GSE3063. A Web site with additional information is available at http://ribonode.ucsc.edu/Sugnet_etal_05/Suppl.

## Abbreviations

CDF: cumulative distribution function
ESE: exonic splicing enhancer
ESS: exonic splicing silencer
EST: expressed sequence tag
ISE: intronic splicing enhancer
ISS: intronic splicing silencers
MAGUK: membrane-associated guanylate kinase
PWM: position-weight matrix
QK: protein quaking
