# Supplementary material for: Unusual Intron Conservation near Tissue-Regulated Exons Found by Splicing Microarrays
Source: PLoS Comput Biol. 2006 Jan 20;2(1):e4. doi: 10.1371/journal.pcbi.0020004 (PMC1331982; doi:10.1371/journal.pcbi.0020004)
Supplement: Supplementary file 1 [file pcbi.0020004.st001.pdf]

Supplementary Table S1. Present-absent detection of alternative splice junction RNA.

Alternative Event	Count	With Probes	Expressed	Alternative	Percent Alternative
Cassette exons	5058	3217	1297	376	29.0%
Alternative 5' splice sites	746	350	136	41	30.1%
Alternative 3' splice sites	1554	530	211	39	18.5%
Mutually Exclusive exons	47	27	15	8	53.3%
Alternative Txn Starts	4262	1537	585	148	25.3%
Alternative Txn Ends	3340	555	189	51	27.0%

Table S1. Expression counts for different types of alternative splicing events. "Count" indicates number of events observed in the EST/mRNA data. "With Probes" indicates events that contain probes on the array. "Expressed" indicates events with at least one isoform that is expressed at false positive rate of 0.1. "Alt" indicates events where at least two isoforms are expressed at false positive rate of 0.1 each. "Percent" indicates the percentage of events with at least one isoform expressed that also had at least one other mutually exclusive isoform expressed.
