# Supplementary material for: Unusual Intron Conservation near Tissue-Regulated Exons Found by Splicing Microarrays
Source: PLoS Comput Biol. 2006 Jan 20;2(1):e4. doi: 10.1371/journal.pcbi.0020004 (PMC1331982; doi:10.1371/journal.pcbi.0020004)
Supplement: Supplementary file 2 [file pcbi.0020004.st002.pdf]

Supplementary Table S2. RT-PCR Testing of Array Predictions

Gene	Junction	Feature	3-Cerebellar Hemisphere	6-Testis	7-Lung	10-Spleen	13-Kidney	16-Stomach	19-Liver	22-Heart	25-Cortex	28-Olfactory Bulb	35-Salivary Gland	38-Small Intestine	41-Esophagus	44-Hind Brain	48-Cerebellum	59-Spinal Cord	60-Skeletal Muscle	65-Colon	69-Mammary Gland
Btd	G6936684@J918253@j_at	inc	NA	+/-	NA	NA	+/-	-/-	+/-	NA	+/-	NA	NA	NA	NA	NA	NA	NA	NA	NA	
Btd	G6936684@J918253@j_at	skip	NA	-/-	NA	NA	-/-	-/-	-/-	NA	-/-	NA	NA	NA	NA	NA	NA	NA	NA	NA	
Chl1	G7091647@J939347@j_at	inc	NA	+/-	NA	NA	NA	NA	NA	NA	-/-	NA	NA	NA	+/+	NA	-/-	NA	NA	NA	
Chl1	G7091647@J939347@j_at	skip	NA	-/-	NA	NA	NA	NA	NA	NA	+/-	NA	NA	NA	-/-	NA	+/-	NA	NA	NA	
Hspa4	G6892948@J912430_RC@j_at	inc	NA	NA	NA	NA	+/-	+/+	NA	NA	NA	NA	NA	NA	NA	NA	NA	NA	NA	NA	
Hspa4	G6892948@J912430_RC@j_at	skip	NA	NA	NA	NA	-/-	-/-	NA	NA	NA	NA	NA	NA	NA	NA	NA	NA	NA	NA	
Ndr3	G7059194@J930125_RC@j_at	inc	NA	NA	NA	NA	NA	NA	NA	NA	+/+	NA	NA	NA	NA	NA	+/+	NA	NA	+/-	
Ndr3	G7059194@J930125_RC@j_at	skip	NA	NA	NA	NA	NA	NA	NA	NA	+/+	NA	NA	NA	NA	NA	+/+	NA	NA	+/-	
Nfia	G6988337@J933418@j_at	inc	NA	NA	+/+	NA	NA	+/-	NA	+/+	+/-	NA	NA	NA	NA	NA	+/+	NA	NA	NA	
Nfia	G6988337@J933418@j_at	skip	NA	NA	-/-	NA	NA	-/-	NA	-/-	-/-	NA	NA	NA	NA	NA	-/-	NA	NA	NA	
Npnt	G7016083@J932308_RC@j_at	inc	NA	NA	+/+	NA	-/-	NA	NA	NA	NA	NA	NA	NA	NA	NA	NA	NA	NA	NA	
Npnt	G7016083@J932308_RC@j_at	skip	NA	NA	-/+	NA	-/+	NA	NA	NA	NA	NA	NA	NA	NA	NA	NA	NA	NA	NA	
Plod1	G7103610@J935013_RC@j_at	inc	NA	NA	-/-	NA	-/-	NA	NA	-/-	NA	NA	NA	NA	NA	NA	-/-	NA	-/-	NA	
Plod1	G7103610@J935013_RC@j_at	skip	NA	NA	+/-	NA	+/-	NA	NA	+/+	NA	NA	NA	NA	NA	NA	+/-	NA	-/-	NA	
Prkaca	G7243535@J944807@j_at	inc	NA	NA	NA	NA	NA	NA	NA	NA	NA	NA	NA	NA	NA	NA	+/+	NA	NA	+/-	
Prkaca	G7243535@J944807@j_at	skip	NA	NA	NA	NA	NA	NA	NA	NA	NA	NA	NA	NA	NA	NA	-/-	NA	NA	-/-	
Surf4	G7105085@J927842_RC@j_at	inc	NA	NA	NA	NA	NA	NA	NA	NA	-/-	NA	NA	NA	NA	NA	-/-	NA	NA	-/+	
Surf4	G7105085@J927842_RC@j_at	skip	NA	NA	NA	NA	NA	NA	NA	NA	+/-	NA	NA	NA	NA	NA	+/-	NA	NA	+/-	
Vldlr	G6995328@J926515@j_at	inc	NA	NA	+/-	NA	+/-	NA	NA	NA	+/-	NA	NA	NA	NA	NA	+/-	NA	NA	NA	
Vldlr	G6995328@J926515@j_at	skip	NA	NA	-/-	NA	-/-	NA	NA	NA	+/-	NA	NA	NA	NA	NA	+/-	NA	NA	NA	
4732496019Rik	G7027520@J931922_RC@j_at	inc	NA	NA	NA	NA	NA	NA	-/-	+/-	+/-	NA	-/-	NA	+/-	NA	+/-	NA	NA	NA	
4732496019Rik	G7027520@J931922_RC@j_at	skip	NA	NA	NA	NA	NA	NA	+/-	+/-	+/-	NA	+/-	NA	+/-	NA	+/-	NA	NA	NA	
Baiap1	G7153846@J939317_RC@j_at	inc	NA	NA	NA	NA	NA	NA	-/-	+/-	+/-	NA	-/-	NA	-/-	NA	+/-	NA	NA	NA	
Baiap1	G7153846@J939317_RC@j_at	skip	NA	NA	NA	NA	NA	NA	+/-	+/-	+/-	NA	+/-	NA	+/-	NA	+/-	NA	NA	NA	
Ndr2	G6912708@J918655_RC@j_at	Ex3 Incl (J2/3)	+/+	-/-	NA	NA	NA	-/-	+/+	+/+	+/+	NA	+/-	-/-	NA	+/+	+/+	+/+	+/+	NA	
Ndr2	G6912708@J918654_RC@j_at	Ex3 Skip (J2/4)	+/+	-/-	NA	NA	NA	-/-	-/-	+/+	+/+	NA	-/-	-/-	NA	+/+	+/+	-/-	-/-	NA	
Clta	G7075177@J932922@j_at	Ex6 Incl (J6/7)	+/-	-/-	NA	-/-	-/-	-/-	NA	-/-	+/-	NA	NA	-/-	-/-	+/-	+/-	NA	+/-	-/-	NA
Clta	G7075177@J932921@j_at	Ex6 skip (J5/7), Ex5 Incl	+/-	-/-	NA	-/-	-/-	-/+	NA	-/-	+/-	NA	NA	-/-	-/-	+/+	+/-	NA	-/-	-/-	
Gaa	G6941378@J914900@j_at	Ex9 Incl (J8/9)	+/+	+/-	+/-	NA	NA	+/-	+/-	+/+	+/+	NA	NA	NA	NA	+/+	+/+	+/-	+/+	NA	+/+
Gaa	G6941378@J914901@j_at	Ex9 Skip (J8/10)	-/-	-/-	-/-	NA	NA	-/-	-/-	-/-	-/-	NA	NA	NA	NA	-/-	-/-	-/-	-/-	NA	
Atp6n1a	G6937984@J914177@j_at	Ex19 Incl (J18/19)	+/+	-/-	-/-	NA	-/-	+/-	NA	-/-	+/+	+/+	NA	-/-	-/-	+/+	+/+	NA	-/-	-/-	-/-
Atp6n1a	G6937984@J914178@j_at	Ex19 Skip (J18/20)	-/-	+/-	+/-	NA	+/-	+/-	NA	+/-	-/-	-/-	NA	+/-	+/-	-/-	-/-	NA	+/+	+/-	+/-
Ssh3bp1 / Abi2	G7125552@J927683_RC@j_at	Ex4 Incl (J4/5)	+/+	+/-	+/+	-/-	NA	+/-	-/-	-/-	+/+	NA	+/-	NA	+/-	+/-	+/+	+/-	-/-	NA	
Ssh3bp1 / Abi2	G7125552@J927684_RC@j_at	Ex4 Skip (J3/5)	-/-	+/+	+/+	-/+	NA	+/+	-/-	-/-	-/-	NA	+/+	NA	+/+	-/-	-/-	+/+	-/-	NA	
Add3	G7037819@J927037@j_at	Ex14 Incl (J14/15)	+/+	+/-	+/-	+/+	+/-	-/+	+/-	-/-	+/+	NA	+/-	-/-	+/-	+/-	-/+	-/-	-/-	+/-	
Add3	G7037819@J927036@j_at	Ex14 Skip (J13/15)	+/+	+/+	+/+	+/+	+/-	-/-	+/-	-/+	+/+	NA	+/-	-/-	+/+	+/+	-/+	-/+	-/+	-/-	
Reps1	G6813886@J909758@j_at	Ex9 Incl (J9/10)	+/+	+/-	+/-	+/-	+/-	-/-	+/-	-/-	+/-	NA	+/-	-/-	+/-	+/-	-/+	+/-	+/-	+/-	
Reps1	G6813886@J909757@j_at	Ex9 Skip (J8/10)	+/-	+/-	+/-	+/-	+/-	-/-	+/-	-/-	+/-	NA	-/-	-/-	+/-	-/-	-/-	+/-	+/-	+/-	
Palm	G6916480@J910611@j_at	Ex8 Incl (J8/9)	+/+	+/-	+/-	+/-	+/-	+/-	-/-	+/-	+/-	NA	+/-	-/-	+/-	+/-	-/-	+/-	-/-	+/-	
Palm	G6916480@J910610@j_at	Ex8 Skip (J7/9)	+/-	+/-	-/-	+/-	+/-	+/-	-/-	-/-	+/+	NA	-/-	-/-	+/-	+/-	-/-	-/-	-/-	+/-	
Mark3	G6899622@J916372@j_at	Ex16 Incl (J15/16)	+/-	-/-	NA	+/-	+/-	+/-	NA	-/-	+/-	NA	NA	NA	NA	+/-	+/-	-/+	+/+	+/-	
Mark3	G6899622@J916373@j_at	Ex16 Skip (J15/17), Ex17 Incl	+/+	+/-	NA	+/-	+/-	+/-	NA	-/-	+/+	NA	NA	NA	NA	+/-	+/+	-/-	-/-	+/-	-/-
Mark3	G6899622@J916376@j_at	Ex17 Incl (J17/18)	+/+	-/-	NA	+/-	+/-	+/-	NA	-/-	+/+	NA	NA	NA	NA	+/-	+/+	-/-	+/-	+/-	-/-
Mark3	G6899622@J916374@j_at	Ex16,17 Skip (J15/18)	-/-	+/-	NA	+/-	+/-	+/-	NA	-/-	-/-	NA	NA	NA	NA	-/-	-/-	-/-	-/-	+/-	
LOC170731 / Mfn2	G7102920@J935009_RC@j_at	Ex3 Incl (J3/4)	-/-	-/-	+/-	NA	-/-	-/-	NA	+/+	-/-	NA	NA	NA	+/-	-/-	NA	NA	+/+	+/-	NA
LOC170731 / Mfn2	G7102920@J935010_RC@j_at	Ex3 Skip (J2/4)	+/+	+/+	+/-	NA	+/-	+/-	NA	-/-	+/+	NA	NA	NA	+/-	+/+	NA	NA	-/-	+/-	
Fgfr1	G7193738@J944099@j_at	Ex3 Incl (J3/4)	+/-	+/-	+/-	NA	NA	+/-	NA	+/-	+/-	+/-	NA	NA	NA	+/-	NA	+/-	+/-	NA	+/-
Fgfr1	G7193738@J944098@j_at	Ex3 Skip (J2/4)	-/-	+/-	+/-	NA	NA	+/-	NA	+/-	-/-	+/-	NA	NA	NA	-/-	NA	+/-	+/-	NA	

Symbols are: (true/predicted values) +/+ : true positives -/- : true negatives +/- : false negatives -/+ : false positives

All genes were used to validate the simple presence-absence test method.
Genes tested in 5 or more different tissues (those below the line) we used to validate the Kruskal-Wallis test for differential tissue expression.
