# Supplementary material for: Unusual Intron Conservation near Tissue-Regulated Exons Found by Splicing Microarrays
Source: PLoS Comput Biol. 2006 Jan 20;2(1):e4. doi: 10.1371/journal.pcbi.0020004 (PMC1331982; doi:10.1371/journal.pcbi.0020004)
Supplement: Supplementary file 3 [file pcbi.0020004.st003.pdf]

Supplementary Table S3. Exons spliced differently in Brain as compared to other tissues.

geneName	knownGene	exon size	mm5.exonPos	flipScore
Smap1	BC006946	81	chr1:24109958-24110039	-1.5063
G6872891@J907741@j_at	G6872891@J907741@j_at	158	chr1:60382871-60383029	2.0164
Fn1	X93167	273	chr1:72037459-72037732	-1.0213
C80879	AF151110	41	chr1:159253712-159253753	2.3923
Pbx1	AF020197	113	chr1:168213213-168213326	-2.8732
Reps1	AK041967	81	chr10:17801476-17801557	-2.0931
Ptprk	L10106	36	chr10:28351604-28351640	-4.235
Ddx50	AK041281	110	chr10:62413421-62413531	0.703
Ank3	L40632	27	chr10:69753810-69753837	-3.661
Nnp1	U79774	83	chr10:78295687-78295770	-0.8485
Ap3d	BC053066	204	chr10:80618285-80618489	1.9553
Slc25a3	BC018161	125	chr10:90892848-90892973	3.9645
4632413K17Rik	BC031768	165	chr10:127219064-127219229	1.6394
BC062928	BC062928	119	chr11:49780121-49780240	-1.3687
2810404F18Rik	AK088090	313	chr11:54379976-54380289	-1.0094
2810404F18Rik	AK088090	154	chr11:54396144-54396298	-1.1094
AA536749	U73200	66	chr11:59384678-59384744	2.7242
Ncor1	U35312	141	chr11:61961892-61962033	1.1394
Map2k4	BC029833	33	chr11:65388280-65388313	-2.1692
Map4k6	AB035697	111	chr11:70221144-70221255	1.9731
Map4k6	AB035697	24	chr11:70221963-70221987	-1.9894
Sept4	X61452	297	chr11:87184982-87185279	-4.7601
Sept4	X61452	119	chr11:87180574-87180693	4.9991
Ppp1r9b	BC029672	104	chr11:94670669-94670773	3.0412
Myst2	BC057102	90	chr11:94962642-94962732	2.624
Psa	U35646	131	chr11:96935018-96935149	1.9679
Atp6v0a1	AF218249	18	chr11:100720581-100720599	-5.4031
AK122484	AK122484	99	chr11:104039804-104039903	1.3227
BC037661	BC037661	189	chr11:106766126-106766315	-1.9259
Wbp2	AK004824	135	chr11:115751674-115751809	1.7081
G6869122@J915373@j_at	G6869122@J915373@j_at	86	chr12:17894317-17894403	1.6395
G6835501@J915591_RC@j_at	G6835501@J915591_RC@j_at	115	chr12:47018382-47018497	-0.7682
Arhgap5	AK041016	145	chr12:47761004-47761149	-2.6146
G6875109@J916256_RC@j_at	G6875109@J916256_RC@j_at	24	chr12:95092530-95092554	-1.9925
Papola	U52197	63	chr12:100552282-100552345	2.1499
Evl	U72519	63	chr12:103436448-103436511	1.5734
Ppp2r5c	AK129042	117	chr12:105321157-105321274	0.9246
Mark3	AF240782	45	chr12:106402416-106402461	-1.9692
Mark3	AF240782	27	chr12:106400984-106401011	3.4598
Kns2	AK031309	51	chr12:106546019-106546070	-1.043
AK122326	AK122326	107	chr12:107776804-107776911	2.4371
Pfkp	BC006926	55	chr13:6386620-6386675	1.781
BC028256	BC028256	117	chr13:100588288-100588405	1.5823
Kif2a	D12644	114	chr13:103720842-103720956	-2.8161
Camk2g	AK078311	69	chr14:17031087-17031156	-0.6215
Camk2g	AK078311	33	chr14:17033114-17033147	-2.4834
4921528E07Rik	BC043475	84	chr14:38257694-38257778	3.4549
Ndrg2	BC012963	42	chr14:44645276-44645318	5.4941
Mbnl2	AK085709	36	chr14:112740624-112740660	-1.7046
2610103J23Rik	AF501309	99	chr15:34176368-34176467	1.5191
Oxr1	AF324899	81	chr15:41822962-41823043	-1.5904
0910001A06Rik	AK028062	39	chr15:64231271-64231310	-0.4118
Pacsin2	AF128535	123	chr15:83735291-83735414	3.2995
Kif21a	BC062896	108	chr15:91347612-91347720	1.8346
Arf3	BC014778	237	chr15:99203610-99203847	-2.127
Pcbp2	X75947	39	chr15:102970638-102970677	1.597
1200013P24Rik	BC004837	125	chr16:3594478-3594603	1.5865
Mgrn1	BC046830	50	chr16:4597749-4597799	-0.7102
Mgrn1	BC046830	66	chr16:4607953-4608019	2.1502
Comt	AF076156	57	chr16:18201307-18201364	1.0226
Ufd1l	U64445	165	chr16:18592931-18593096	0.5995
Eif4a2	AK088669	90	chr16:22955496-22955586	-1.9615
Eif4a2	AK088669	111	chr16:22956344-22956455	3.5343
App	X59379	168	chr16:85426246-85426414	5.9513
Ttc3	AB008516	43	chr16:95093583-95093626	2.1745
G6966427@J922632_RC@j_at	G6966427@J922632_RC@j_at	135	chr17:5980315-5980450	3.005
RFWD1	BC008598	117	chr17:23110611-23110728	-0.5236
Btbd9	BC057897	212	chr17:29132782-29132994	-2.8068
Brd2	AF045462	92	chr17:32614795-32614887	0.9679
Bat3	BC026647	54	chr17:33635450-33635504	-0.6028
AK011541	AK011541	110	chr17:55028765-55028875	2.1152
Pja2	AF493070	186	chr17:62653417-62653603	2.7773
Epb4.1l3	AF152247	36	chr17:67705634-67705670	-2.5414
Epb4.1l3	AF152247	123	chr17:67715637-67715760	-3.2996
Epb4.1l3	AF152247	117	chr17:67719364-67719481	-5.3363
G6995877@J924815@j_at	G6995877@J924815@j_at	67	chr18:25033024-25033091	-1.3125
Bin1	U86405	129	chr18:32883311-32883440	-3.1665
AK029322	AK029322	80	chr18:37084598-37084678	1.9459
Rnf14	AF249667	110	chr18:38740624-38740734	1.4324
Mark2	AK045329	162	chr19:6995224-6995386	-0.9992
AY427822	AY427822	57	chr19:7181237-7181294	-1.5817
Coro1b	AF143956	203	chr19:3937811-3938014	0.8554
5730453I16Rik	AK053375	66	chr19:9631211-9631277	1.2522
G6981819@J926487@j_at	G6981819@J926487@j_at	290	chr19:22990528-22990818	-0.7817
BC056199	BC056199	106	chr19:26046719-26046825	-1.822
Vldlr	U06670	123	chr19:26512799-26512922	2.0765
Vldlr	U06670	84	chr19:26523084-26523168	3.192

Atad1	BC029085	175	chr19:32017100-32017275	-1.9365
AK122487	AK122487	168	chr19:39657509-39657677	2.0866
AK122487	AK122487	90	chr19:39731986-39732076	1.8635
Stk2	AK122218	93	chr19:46972197-46972290	2.4021
Ablim1	AK122196	141	chr19:56435308-56435449	3.6529
G7125552@J927679_RC@j_at	G7125552@J927679_RC@j_at	87	chr2:22910189-22910276	1.9067
G7125552@J927684_RC@j_at	G7125552@J927684_RC@j_at	15	chr2:22923219-22923234	-4.1268
Surf4	M63114	77	chr2:26885629-26885706	1.0233
BC027791	BC027791	15	chr2:29974179-29974194	-4.3725
BC023143	BC023143	84	chr2:35226810-35226894	2.0632
Orc4l	AJ003140	68	chr2:48869256-48869324	-1.0344
Fnbp3	AF135439	54	chr2:53119253-53119307	2.767
Tlk1	BC051641	69	chr2:70707497-70707566	-1.5645
Dncic2	AF063231	60	chr2:71127814-71127874	-3.7508
Atf2	BC042210	97	chr2:73747960-73748057	-1.6152
6330583M11Rik	BC002138	41	chr2:150964842-150964883	1.6095
Samhd1	BC067198	123	chr2:157164669-157164792	1.0726
3632413B07Rik	AB093284	80	chr2:165850276-165850356	2.5736
AK129214	AK129214	175	chr2:168267717-168267892	1.1603
Dpm1	AK004834	69	chr2:168276077-168276146	1.3136
Bcas1	AY219233	138	chr2:170447306-170447444	-7.0571
Gnas	AF107848	45	chr2:174404211-174404256	-3.5742
Dnajc5	BC012268	53	chr2:181209352-181209405	1.8744
4932431F02Rik	AK048286	75	chr3:90460446-90460521	1.6553
4732496O19Rik	AF213258	91	chr3:103947167-103947258	-2.2145
Sars1	AK082793	72	chr3:108807895-108807967	-1.3026
Camk2d	AK039076	89	chr3:127323009-127323098	2.1947
Rap1gds1	BC011279	147	chr3:139541762-139541909	3.0401
AF133818	AF133818	109	chr3:158152010-158152119	-2.0635
Rod1	AK088560	34	chr4:58814247-58814281	0.5474
Eps15	L21768	182	chr4:107728425-107728607	-2.2761
G7094107@J933920_RC@j_at	G7094107@J933920_RC@j_at	33	chr4:116563652-116563685	-3.4057
2210021A15Rik	AB041805	48	chr4:116757166-116757214	0.9563
BC019977	BC023677	111	chr4:124608837-124608948	-1.9185
Rbbp4	U35141	148	chr4:127704812-127704960	-1.2306
G7091230@J934506@j_at	G7091230@J934506@j_at	129	chr4:136511733-136511862	-0.8612
BC047531	BC047531	116	chr4:136638436-136638552	-1.3999
Mfn2	AF384100	83	chr4:145788869-145788952	7.0757
Kif1b	AF090190	18	chr4:147151286-147151304	-4.2161
Clstn1	BC053843	57	chr4:147527599-147527656	-2.764
Cacna2d1	U73483	57	chr5:14682361-14682418	6.2346
Cacna2d1	U73483	21	chr5:14701647-14701668	-5.6941
Ctbp1	BC042425	193	chr5:31717369-31717562	-0.9477
Add1	AF096839	279	chr5:33043848-33044127	0.9955
Add1	AF096839	87	chr5:33066510-33066597	-0.9116
AK122316	AK122316	135	chr5:61241764-61241899	-0.8786
Hip2	BC002013	94	chr5:64202917-64203011	-0.8238
Hip2	BC002013	83	chr5:64218413-64218496	-1.1829
Hnrpd	BC049098	107	chr5:97285513-97285620	1.1623
BC054358	BC054358	336	chr5:97699337-97699673	-0.7765
Chchd3	AK003519	94	chr6:32673648-32673742	-2.2083
Immt	AK038129	96	chr6:72080716-72080812	-2.6188
Copg	AF187079	53	chr6:88225742-88225795	-0.6794
Mgll	AJ316580	84	chr6:89151028-89151112	5.798
Baiap1	AK032350	89	chr6:94036663-94036752	-2.6359
AF340029	AF340029	67	chr6:120010274-120010341	-1.8882
Drpla	BC053051	144	chr6:125361242-125361386	-1.4042
Csda	BC048242	207	chr6:131684086-131684293	-2.222
Kras2	BC004642	124	chr6:145385064-145385188	1.361
Ppp5c	BC003744	242	chr7:8708333-8708575	-1.7162
Pld3	AF026124	143	chr7:16480054-16480197	-1.1337
Sirt2	BC021439	47	chr7:17736535-17736582	0.608
Usf2	U01663	201	chr7:19928296-19928497	-0.9324
BC038157	BC038157	176	chr7:51392314-51392490	0.5516
Mfge8	BC003892	111	chr7:66217550-66217661	2.2604
Arrb1	AK052588	24	chr7:86871505-86871529	-0.5712
Tacc2	AB041546	126	chr7:118196540-118196666	1.311
BC017520	BC017520	160	chr7:120649119-120649279	-1.4541
Fgfr1	BC010200	267	chr8:24332778-24333045	-3.296
Gpsn2	BC019984	52	chr8:82837902-82837954	-2.3189
Nfix	Y07688	148	chr8:83989978-83990126	-1.1124
Nfix	Y07688	123	chr8:83997757-83997880	-0.4703
BC058645	BC058645	154	chr8:103795341-103795495	2.0379
AK032084	AK032084	145	chr8:122389607-122389752	-0.9194
Mrpl4	AB049634	24	chr9:20931996-20932020	0.9132
E430039K05Rik	AK046492	115	chr9:21997235-21997350	-0.5833
Aplp2	U15571	36	chr9:31135756-31135792	-4.6848
2300004C15Rik	BC016456	213	chr9:58118988-58119201	-0.6564
Tcf12	BC037097	72	chr9:72189645-72189717	-2.4244
Senp6	BC061480	21	chr9:80502690-80502711	-1.533
Azi2	AK030076	47	chr9:118031515-118031562	2.6125
Cask	Y17138	69	chrX:11884093-11884162	-1.8176
AK083192	AK083192	81	chrX:125750603-125750684	1.1064
AK031374	AK031374	108	chrX:141723672-141723780	1.8859
