# Supplementary material for: Unusual Intron Conservation near Tissue-Regulated Exons Found by Splicing Microarrays
Source: PLoS Comput Biol. 2006 Jan 20;2(1):e4. doi: 10.1371/journal.pcbi.0020004 (PMC1331982; doi:10.1371/journal.pcbi.0020004)
Supplement: Supplementary file 4 [file pcbi.0020004.st004.pdf]

Supplementary Table S4. Exons spliced differently in Muscle as compared to other tissues.

geneName	knownGene	exon size	mm5.exonPos	flipScoreAllTissues
Ndufs1	BC006660	104	chr1:63564700-63564804	1.4101
Ndufs2	BC016097	58	chr1:171306504-171306562	-2.3767
Slc25a3	BC018161	125	chr10:90892848-90892973	-4.3711
Ccm2	BC029157	174	chr11:6464752-6464926	1.1595
Pdk2	AF267660	114	chr11:94698987-94699101	-1.4988
Atp6v0a1	AF218249	18	chr11:100720581-100720599	6.2499
Nkiras1	AK028539	258	chr14:14949878-14950136	-1.5397
Mbnl2	AK085709	95	chr14:112741990-112742085	1.795
Tsc2	U39818	129	chr17:23202122-23202251	-1.6772
Mapk14	U10871	45	chr17:27286394-27286439	1.5281
2410001H17Rik	AK009263	45	chr19:3919500-3919545	0.566
Vldlr	U06670	123	chr19:26512799-26512922	-2.2546
AK122487	AK122487	60	chr19:39635743-39635803	-4.0079
AK122487	AK122487	168	chr19:39657509-39657677	-2.0285
AK122487	AK122487	90	chr19:39731986-39732076	-2.5683
Zfp106	BC025424	2169	chr2:120315086-120317255	-1.8328
Idh3b	BC009022	171	chr2:130028578-130028749	7.038
Fxr1h	BC019139	92	chr3:33862715-33862807	-3.7742
Capzb	U10406	113	chr4:137742645-137742758	-3.056
Sdhb	BC051934	134	chr4:139422796-139422930	-1.555
Mfn2	AF384100	83	chr4:145788869-145788952	-5.8237
Art3	AJ311773	30	chr5:91238008-91238038	-4.7778
Mpp6	AF161181	42	chr6:50126528-50126570	-4.4997
Csda	BC048242	207	chr6:131684086-131684293	1.4625
Phkb	BC053105	92	chr8:85123611-85123703	1.8725
Idh3a	BC049956	102	chr9:54800770-54800872	1.8845
Azi2	AK030076	47	chr9:118031515-118031562	-3.9122
AK004035	AK004035	91	chrX:65847312-65847403	1.7087
